# Revealing the invisible: a new imaging paradigm for α-synuclein pathology in Parkinson's disease

**DOI:** 10.3389/fradi.2026.1730757

**Published:** 2026-01-30

**Authors:** Jian Meng, Xingbo Li

**Affiliations:** 1 Department of Radiology, The 80th Group Army Hospital of the PLA Army, Weifang, Shandong, China; 2 Hand and Foot Surgery Center, The 80th Group Army Hospital of the PLA Army, Weifang, Shandong, China

**Keywords:** ASA-PD imaging technique, neurodegenerative biomarkers, Parkinson's disease, single-molecule fluorescence microscopy, α-synuclein oligomers

## Introduction

A recent study published in *Nature Biomedical Engineering* reported the development of Advanced Sensing of Aggregates-Parkinson's Disease (ASA-PD), a single-molecule fluorescence imaging and analysis technique that enables large-scale nanoscale mapping of *α*-synuclein assemblies in post-mortem human brain tissue (1). By systematically imaging and analyzing over a million nanoscale assemblies, the authors revealed a distinct subpopulation of bright, protease-resistant oligomers enriched in diseased tissue. These findings offer direct spatial and quantitative evidence linking oligomeric species to human PD pathology, bridging a gap that has long separated mechanistic models from clinical neuropathology.

The histopathological definition of Parkinson's disease (PD) has long rested on the presence of Lewy bodies and Lewy neurites, which are intraneuronal inclusions composed of fibrillar aggregates of α-synuclein (2). These structures define Braak staging and correlate with disease severity. Yet growing evidence suggests that the early pathogenic events occur at a much smaller scale, involving oligomeric *α*-synuclein assemblies rather than mature fibrils (3, 4). These nanoscale intermediates are thought to seed aggregation, disrupt membranes, and induce cellular toxicity, but until now their direct visualization in human tissue has been limited.

The current landscape of *in vivo* imaging includes efforts in both magnetic resonance and nuclear medicine. Chemical Exchange Saturation Transfer Magnetic Resonance Imaging (CEST-MRI), an advanced MRI technique, is being explored as an indirect biomarker for synucleinopathies (5). CEST-MRI detects low-concentration molecules by sensitizing the water signal to exchangeable protons on tissue components like proteins or metabolites. Studies have shown its potential to distinguish PD patients from controls by detecting changes in amide proton transfer (APT) signals in structures like the substantia nigra and striatum, which is thought to reflect regional differences in the concentration of aggregated or misfolded proteins (6).

Simultaneously, the development of specific Positron Emission Tomography (PET) Tracers that bind directly to α-synuclein aggregates is viewed as a critical goal for clinical PD imaging. Although this task is complicated by the target's low abundance and conformational heterogeneity, a major translational breakthrough was recently reported with tracers such as [18F]C05-05, which achieved the first successful visualization of α-synuclein deposits *in vivo* in the midbrain of PD patients. Notably, the signal intensity observed with this tracer correlated with clinical motor severity, confirming its potential as a disease biomarker (7).

**Figure 1:**
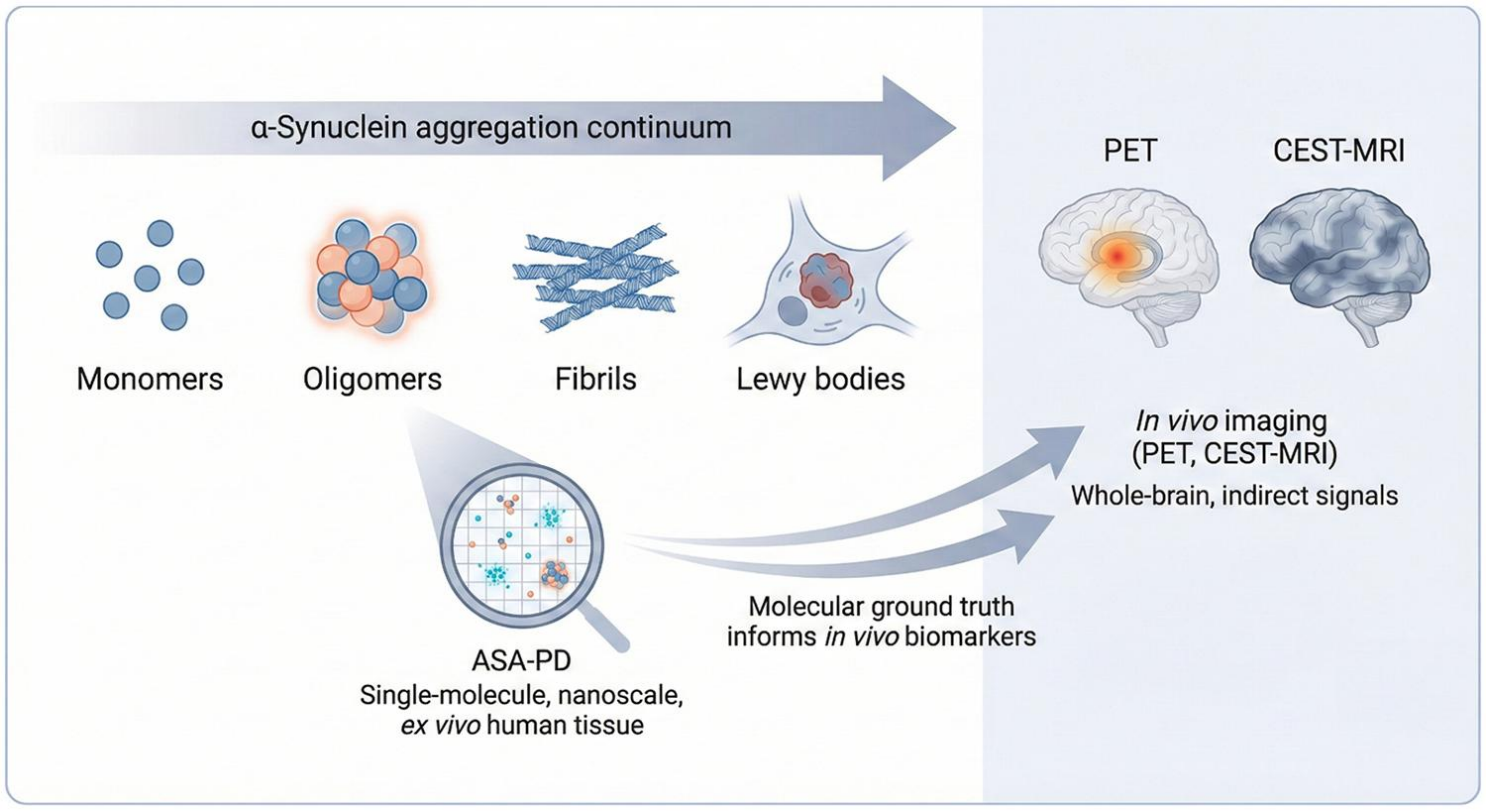
Conceptual framework linking α-synuclein aggregation, ASA-PD, and *in vivo* imaging. α-Synuclein pathology in Parkinson's disease progresses along a continuum from monomeric species through oligomeric intermediates to fibrils and mature Lewy bodies. Emerging evidence suggests that oligomeric assemblies represent early, biologically active pathogenic species. ASA-PD enables single-molecule, nanoscale visualization and quantification of these oligomeric α-synuclein assemblies in *ex vivo* human brain tissue, providing high-resolution molecular ground truth. In contrast, *in vivo* imaging modalities such as PET and CEST-MRI capture whole-brain, indirect signals of pathology at the macroscopic scale. By defining disease-specific oligomeric signatures and their spatial organization, ASA-PD offers a mechanistic bridge between molecular pathology and the interpretation and development of *in vivo* imaging biomarkers.

The power of the ASA-PD study discussed in this article lies in its ability to validate and inform these *in vivo* clinical efforts at the molecular level. The nanoscale structural and quantitative data regarding specific oligomeric signatures obtained from ASA-PD could directly influence the rational design and improve the selectivity of the next generation of CEST-MRI agents or α-syn-targeted PET tracers, thereby accelerating the transition of molecular-level insights into clinical diagnostic tools. To aid conceptual clarity, a schematic overview of α-synuclein aggregation states and the specific positioning of the ASA-PD approach within the broader imaging landscape is provided (Figure 1).

## Methodological advances and key findings

The ASA-PD imaging technique integrates autofluorescence quenching, high-numerical-aperture single-molecule fluorescence microscopy, and computational image analysis to detect nanoscale α-synuclein assemblies with exceptional sensitivity. Autofluorescence suppression using Sudan Black B, combined with high-resolution optics, allowed visualization of puncta far below the diffraction limit without compromising coverage.

Across approximately 12,000 images from 30 tissue sections, ASA-PD detected around 125,000 large aggregates and 1.26 million nanoscale puncta. Importantly, a bright subpopulation accounting for about 10% of assemblies in PD tissue but only 0.3% in controls was identified. These bright puncta correspond to nanoscale assemblies enriched in PD and align with biochemically defined fractions shown in orthogonal assays to be seed competent and resistant to Proteinase K digestion. Spatial mapping revealed preferential clustering around neurons, astrocytes, and microglia, indicating selective enrichment in biologically relevant cellular niches.

The scale of the imaging approach provided the statistical resolution needed to detect disease-linked shifts in nanoscale aggregate populations. In parallel, multiplexed immunostaining anchored these assemblies within their precise anatomical and cellular context, enabling meaningful biological interpretation.

This work aligns with an expanding literature implicating oligomeric *α*-synuclein as an initiating factor in PD. A 2025 study demonstrated that oligomer injection into rodent brains produced early synaptic dysfunction and inflammation before fibrillar pathology emerged (8). Another report visualized pore formation by oligomers on membranes, elucidating a plausible mechanism of toxicity (9). In parallel, amplification assays such as RT-QuIC have detected seeding activity in the cerebrospinal fluid of individuals at prodromal disease stages, underscoring their diagnostic potential (10).

Together, these studies support a model in which oligomers represent a biologically active and clinically relevant phase of α-synuclein aggregation (11). The ASA-PD imaging technique extends this framework by anchoring these molecular species to their spatial distribution in human tissue, bridging experimental models with neuropathological reality.

## Strengths and limitations of the oligomer mapping approach

A key strength of the study lies in its combination of nanoscale sensitivity and large tissue coverage, enabling precise quantification of rare but disease-relevant oligomer populations. The integration of multiplexed staining allowed these structures to be interpreted in their tissue microenvironment, revealing nonrandom patterns of cellular association.

Several limitations are worth noting. Fluorescence brightness serves only as a proxy for aggregate size below the diffraction limit, necessitating future correlation with electron or super-resolution microscopy. The exclusive focus on pS129 α-synuclein may underrepresent other oligomeric species. Anatomical coverage was limited to the anterior cingulate cortex, and oligomer patterns likely vary across brain regions and disease stages. Finally, despite its throughput, scaling to whole-brain mapping will require further automation and technical refinement. These are natural next steps for extending the impact of the work.

## Diagnostic and therapeutic implications

Direct visualization of oligomeric *α*-synuclein in human tissue establishes a framework for developing tissue-based biomarkers. If specific oligomer signatures correlate with disease stage or clinical trajectory, they could guide both diagnosis and patient stratification. Over time, these molecular signatures may inform the development of *in vivo* imaging tracers, potentially enabling the detection of pathogenic conformers in the living brain.

Therapeutically, the findings emphasize the importance of intervening upstream of Lewy body formation. Promising strategies may include stabilizing native α-synuclein conformers, inhibiting oligomer formation, or modifying the microenvironment that facilitates their accumulation. Their preferential enrichment near neurons and glia indicates that tissue context instead of just protein chemistry may be a critical driver of pathology, broadening the spectrum of potential targets.

Because similar oligomeric intermediates have been implicated in Alzheimer's disease, TDP-43 proteinopathies, and Huntington's disease, applying comparable mapping approaches across diseases could reveal shared mechanisms of early protein aggregation.

## Discussion

The ASA-PD imaging technique represents an important advance in the visualization of pathological protein assemblies in PD. For the first time, oligomeric α-synuclein has been mapped at nanoscale resolution across large human tissue regions, revealing a disease-specific subpopulation that may drive early pathogenic processes. Importantly, this work reframes α-synuclein pathology not as a static endpoint defined by Lewy bodies, but as a continuum of molecular states with distinct biological roles. Compared with conventional neuropathological approaches that primarily identify late-stage fibrillar inclusions such as Lewy bodies, ASA-PD enables direct visualization and quantification of early oligomeric α-synuclein assemblies at the nanoscale. In contrast to *in vivo* imaging modalities such as PET or CEST-MRI, which provide macroscopic or indirect signals constrained by sensitivity and specificity, ASA-PD offers molecular-resolution ground truth in human tissue. This positions ASA-PD as a critical bridge between structural pathology and emerging *in vivo* imaging biomarkers.

A key translational implication of the ASA-PD study is the opportunity it presents to refine the development of *in vivo* imaging biomarkers, which remain indispensable for clinical diagnosis and monitoring of PD progression. While methods such as CEST-MRI and the use of novel PET tracers are advancing rapidly, they face common challenges, notably the low concentration of pathological α-synuclein aggregates and the stringent requirement for high target specificity. The ASA-PD findings directly address the need for molecular ground truth. By identifying a distinct, disease-specific population of nanoscale *α*-synuclein oligomers, this *ex vivo* approach provides a high-fidelity structural signature that can guide future tracer development. Specifically, knowledge of the precise conformational and environmental characteristics of these pathogenic aggregates could inform the rational design of PET radioligands, moving beyond current pan-synuclein tracers toward agents that selectively bind to the PD-specific fold. Similarly, this molecular detail can enhance the interpretation of subtle signal changes observed in CEST-MRI studies, such as the differential amide proton transfer signals detected in the substantia nigra of PD patients, by linking these macroscopic imaging alterations directly to the presence and clustering of specific, toxic *α*-synuclein species. Ultimately, the successful integration of high-resolution structural pathology from techniques like ASA-PD with *in vivo* imaging data is essential for validating disease-modifying therapies that specifically target *α*-synuclein. By defining disease-specific oligomeric signatures and their spatial organization, ASA-PD provides a molecular reference framework that can be used to interpret, validate, and refine *in vivo* imaging signals obtained from PET and MRI-based approaches.

Moving forward, key questions include how oligomer populations evolve over time, what determines their conversion to seed-competent forms, and how their microenvironment influences these transitions. Addressing these questions will require integrating nanoscale mapping with structural, molecular, and clinical datasets. Such efforts could ultimately reshape both biomarker strategies and therapeutic development for PD and related disorders.
